# Chirality of Modern Antidepressants: An Overview

**DOI:** 10.15171/apb.2017.061

**Published:** 2017-12-31

**Authors:** Monica Budău, Gabriel Hancu, Aura Rusu, Melania Cârcu-Dobrin, Daniela Lucia Muntean

**Affiliations:** ^1^Department of Pharmaceutical Chemistry, Faculty of Pharmacy, University of Medicine and Pharmacy from Tîrgu Mureş, Tîrgu Mureş, Romania.; ^2^Department of Analytical Chemistry and Drug Analysis, Faculty of Pharmacy, University of Medicine and Pharmacy from Tîrgu Mureş, Tîrgu Mureş, Romania.

**Keywords:** Antidepressants, Selective serotonin reuptake inhibitor (SSRI), Selective serotonin and norepinephrine reuptake in, Stereoselectivity, Chirality

## Abstract

The majority of modern antidepressants (selective serotonin reuptake inhibitors and selective serotonin and norepinephrine reuptake inhibitors) have one or two centers of asymmetry in their structure; resulting in the formation of enantiomers which may exhibit different pharmacodynamic and pharmacokinetic properties. Recent developments in drug stereochemistry has led to understanding the role of chirality in modern therapy correlated with increased knowledge regarding the molecular structure of specific drug targets and towards the possible advantages of using pure enantiomers instead of racemic mixtures. The current review deals with chiral antidepressant drugs; presenting examples of stereoselectivity in the pharmacological actions of certain antidepressants and their metabolites and emphasizing the differences between pharmacological actions of the racemates and pure enantiomers.

## Introduction

Depression (major depressive disorder or clinical depression) is a common but serious mood disorder that affects a person's behavior, feelings, thoughts and sense of well-being. Nowadays depression is the most common mental health problems worldwide; it is estimated that up to 20% of the population will suffer from depressive episodes at least once in their lives.^1^

Most frequently used antidepressants drugs potentiate, the actions of norepinephrine and/or serotonin in the brain. Before 1990, antidepressant treatment consisted primarily in the administration of tricyclic antidepressants; drugs which presented numerous side-effects due to the binding to multiple unrelated receptors (H1-histaminic, α1-adrenergic, muscarinic cholinergic receptors). The introduction in therapy in the late 1980s of the first selective serotonin reuptake inhibitor (SSRI), fluoxetine, is considered the “breakthrough” in the modern treatment of depression.^2^

The need for a large range of antidepressants with differing pharmacological profiles is driven by the diversity of clinical manifestations observed during depressive episodes.^3^

The SSRI class, which includes fluoxetine, citalopram, sertraline, paroxetine and fluvoxamine; has become the most frequently prescribed antidepressant treatment because of certain advantages over the tricyclic antidepressants in terms of tolerability, safety and ease of dosing. The SSRIs block the reuptake of serotonin, resulting in increased concentrations of the neurotransmitter in the synaptic cleft and, and to greater postsynaptic neuronal activity.^4^

Selective serotonin and norepinephrine reuptake inhibitors (SSNRIs), like venlafaxine and duloxetine are also used in the modern treatment of depression and may be effective in treating depression in patients in whom SSRIs are ineffective.^5^

SSRIs and SSNRIs are among the most interesting pharmaceutical substances from stereochemistry point of view, as with the exception of fluvoxamine all substances currently used in therapy are optically active containing at least a chiral center in their structure; also in the class of modern antidepressants we can find substances which are marketed as racemic mixtures (fluoxetine, venlafaxine), optically pure enantiomers (sertraline, paroxetine, duloxetine) or experienced “chiral switch” (citalopram).^6^

In an achiral environment enantiomers of a chiral drug have identical physical and chemical properties; but in a chiral environment, one enantiomer may display different chemical and pharmacological behavior than the other enantiomer. The human body is composed of chiral elements, consequently is the most complex chiral selector, which explains different or varying pharmacological responses to different enantiomers.^7^

Usually the desired pharmacological effect is limited to only one of the enantiomers, called eutomer, while the other one, called distomer, could be inactive, less active or in some cases could be responsible for the adverse effects. Sometimes for a given chiral drug, it is appropriate to consider the enantiomers as separate drugs with different properties unless proven otherwise.^8^

The enantiomers of a chiral drug can be identified taking in consideration their absolute configurations or their optical rotations. The (R)- and (S)-nomenclature according to Cahn, Ingold and Prelog (CIP) rules of priority characterize the absolute configuration of a certain stereogenic center, in the case of the substances presented in this review a tetracoordinated carbon atom substituted by four different substituents. The prefixes (+)– and (–)-characterize the rotation of polarized light to the right or to the left, and therefore, describes only a physicochemical property of the enantiomers. From a stereochemical point of view, only the CIP nomenclature is configurationally descriptive, consequently is recommended to be used by IUPAC.^9^

The present article reviews the stereochemical aspects of modern antidepressants, emphasizing on potential biological and pharmacologic differences between the enantiomers and highlighting the potential advantages of using pure enantiomers.

### Selective serotonin reuptake inhibitor (SSRI)

SSRIs, whose model drug is fluoxetine, are the most frequently used class of second-generation antidepressant drugs and are considered the drugs of first choice in the treatment of depression; having a similar therapeutic efficacy, but a more favorable toxic- and side-effect profile in comparison with traditional tricyclic antidepressants.^10^

### Fluoxetine

**Figure 1:**
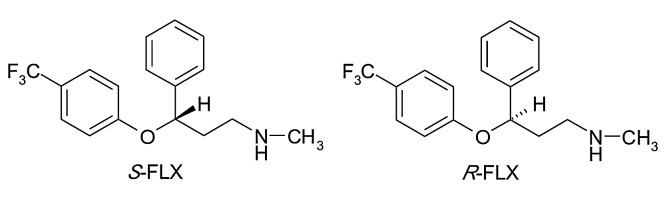
Structure of FLX enantiomers

Fluoxetine (*R,S*- N-methyl-3-phenyl-3-[4-(trifluoromethyl)phenoxy]propan-1-amine) (FLX), was the first SSRIs introduced in therapy and is used for the treatment of major depressive disorder, panic disorder, obsessive–compulsive disorder, premenstrual dysphoric disorder and nervous bulimia.^11^ It has a chiral center in its structure and is used in therapy as a racemic mixture. The chemical structure of FLX enantiomers are presented in Figure 1.

Both *R*-FLX and *S*-FLX are similarly effective *in vitro* at blocking serotonin reuptake, but everything changes *in vivo* as they are metabolized differently.

FLX is metabolized stereoselectively through cytochrome P450 enzyme system in the liver by N-demethylation; to an active chiral metabolite, norfluoxetine (NFLX), which is also a potent SSRI. NFLX has a very similar pharmacologic profile to FLX and reaches similar plasma levels; consequently, it contributes to the pharmacologic effect of FLX treatment.^12,13^

*R*-FLX and *S*-FLX have similar potencies as SSRI, but *S*-NFLX is a more potent SSRI than *R*-NFLX. Furthermore, in patients treated with *R,S*-FLX, plasma concentration levels of S-NFLX have been found to be greater than those of R-NFLX.^12^

*R*-FLX and *S*-FLX have different metabolic rates, as the clearance of *R*-FLX is about four times greater than the one of *S*-FLX.^14^ The elimination half-life of *S*-FLX and *S*-NFLX are affected to a greater extent than the one of the *R*-enantiomers by the variability in CYP2D6 activity.^13,15^

*In vitro* studies on the stereoselective metabolism of FLX and NFLX show that in addition to CYP2D6, CYP2C9 and to a minor degree CYP2C19 are also implicated in FLX metabolism by N-demethylation, with preference towards formation of *R*-NFLX. On the other hand, CYP2D6 displays higher activity towards *S*-FLX than *R*-FLX.^15,16^

The use of *R*-FLX was expected to result in less variable plasma levels of FLX and NFLX than observed with *R,S*-FLX administration. Consequently, clinical trials were undertaken in order to evaluate the safety and efficiency of *R*-FLX. However, in phase II clinical studies, administration of high doses of *R*-FLX, led to a small but statistically significant prolongation of cardiac repolarization, and the studies were stopped.^8,11^*S*-FLX was also evaluated in clinical studies for the prophylaxis of migraine, but has not received FDA approval at this date.^11^

### Citalopram

Citalopram (*R,S*-1-(3-(dimethylamino)propyl)-1-(4-fluorophenyl)-1,3-dihydroisobenzofuran-5-carbonitrile) (CIT) is an SSRI antidepressant used in the treatment of major depression, obsessive-compulsive disorder and panic disorder.^17^

**Figure 2:**
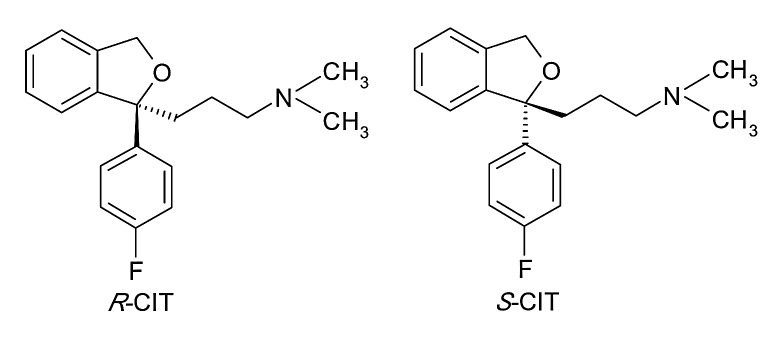
Stereoselective FLX metabolism

It was initially used as a racemic drug, but differences between the pharmacological potencies of the enantiomers led to the “chiral switch” to *S*-citalopram or escitalopram.^18^ “Chiral switch” is a term used to characterize the replacement of a racemate of drug which has already been approved and marketed by a pure enantiomer.^19^ Currently CIT is the only antidepressant with both racemic and pure enantiomer formulation on the market. The chemical structure of CIT enantiomers is presented in Figure 2.

*S*- CIT is primarily responsible for the antagonism of serotonin reuptake, *R*-CIT being 30-fold less potent.^20^ Following administration of *R,S*-CIT, the plasma concentration of *S*-CIT is approximately one third of those of the total drug, though it is not clear if the more rapid elimination of *S*-CIT is due to stereoselective actions of cytochrome P450 enzymes in the liver.^21^

As with FLX, CIT metabolism by demethylation, leads to a active metabolite, desmethylcitalopram (DCIT), which in the case of *S*-enantiomer is approximately six fold less potent than the parent drug; however the *R*-DCIT of the metabolite is approximately four fold more potent than *R*-CIT. DCIT is further N-demethylated to didesmethylcitalopram (DDCIT) which has weak SRI activity and reaches lower plasma concentrations than the parent drug and its main metabolite.^18,22^

*S*-CIT administration presents several advantages over *R,S*-CIT including increased potency, administration of smaller doses and the avoidance of adverse effects attributable to the *R*-CIT; being a good example of the potential benefits of “chiral switch”.^19^

Based on the quantity of administered *S*-CIT (either as *S*-CIT or *R,S*-CIT), it was expected that a given dose of *S*-CIT would be equivalent to twice the dose of *R,S*-CIT.^23^ However the results of clinical studies showed numerically superior results when using *S*-CIT.^24^ Several evidence indicates an effect of *R*-CIT on the association of *S*-CIT with the high affinity primary site, and on its dissociation from the serotonin transporter, via an allosteric mechanism; which could explain a possible antagonism of *R*-CIT on the effect of *S*-CIT. This serotonin dual action in binding to two sites on the serotonin transporter (primary site and allosteric site) could be responsible for a longer binding, and therefore greater inhibition of the serotonin transporter by *S*-CIT, as *S*-CIT can be considered an allosteric serotonin reuptake inhibitor.^25^

Furthermore, *R*-CIT is a substrate for CYP P4502D6 and hence genetic polymorphism and variability in drug concentrations would be avoided by the marketing of the *S*-CIT.^26^

The results of randomized, double-blind placebo, controlled clinical trials showed that *S*-CIT has greater efficiency than *R,S*-CIT at doses predicted to be equivalent and equal efficiency to *R,S*-CIT at doses that led to fewer discontinuation of medication.^27^ Clinical trials also suggested that *S*-CIT may have an earlier onset of action than *R,S*-CIT, and that the pure enantiomer appears to be better tolerated than *R,S*-CIT.^28^ However, there is currently no evidence that patients suffering from major depression who are responding well to *R,S*-CIT benefit from switching to *S*-CIT.

### Sertraline

Sertraline (*(+)-cis-(1S,4*S)-N-methyl-4(3,4-dichlorophenyl)-1,2,3,4-tetrahydro-1-naphthalenamine) (SER), is an SSRI used to treat major depression as well as panic, obsessive-compulsive and social anxiety disorders.^29^

**Figure 3:**
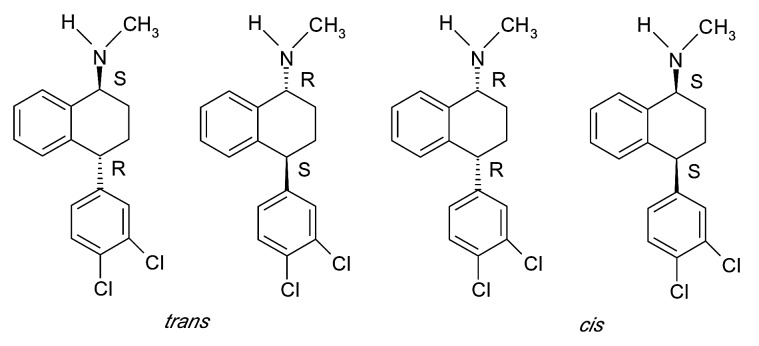
Structure of CIT enantiomers

It contains two centers of chirality in its structure, and is marketed as a pure enantiomer. The stereoisomer used in therapy has a *(+)-cis-(1S,4*S) configuration. The chemical structure of SER diastereomers is presented in Figure 3.

In the case of *trans* isomers, the *(+)-1R,4S*-enantiomer is a potent inhibitor of serotonin, dopamine and norepinephrine while the *(-)-1S,4R*-enantiomer is more selective for inhibition of norepinephrine. In the case of *cis* isomers a separation of activity occurs with the (+)-*1S,4S*-enantiomer which retains a potent serotonin uptake inhibition activity. In therapy the (+)-*cis-1S,4S* is used because is more selective in the inhibition of serotonine uptake even if it is two fold less potent than the *(+)-tran-1R,4S*-enantiomer.^30^

### Paroxetine

Paroxetine ((-)-trans-(3S,4R)-3-(1,3-benzodioxol-5-yloxymethyl)-4-(4-fluorophenyl)piperidine) (PAR) ), is an SSRI used to treat major depressive disorder, social anxiety disorder, panic disorder, obsessive-compulsive disorder, generalized anxiety disorder, posttraumatic stress disorder and premenstrual dysphoric disorder.^31^

**Figure 4:**
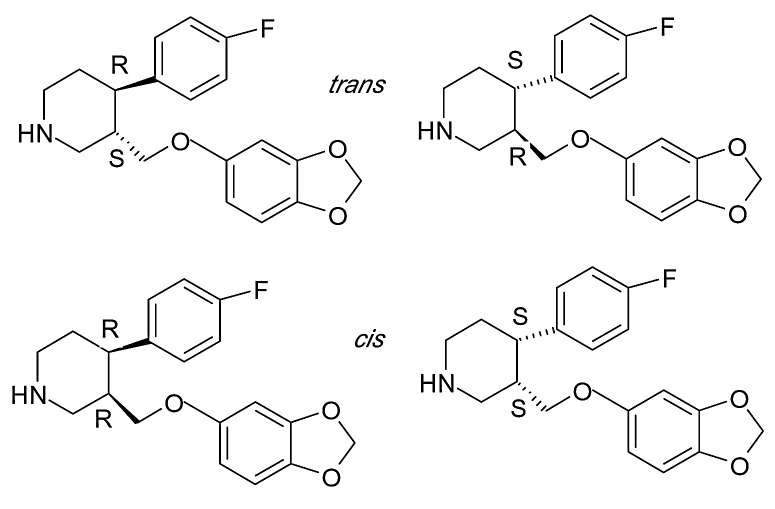
*S*-CIT metabolism

It contains two centers of chirality in its structure, and is marketed as a pure enantiomer. The stereoisomer used clinically has an absolute configuration of *(-)-trans-(3S,4R)*. The chemical structure of PAR diastereomers is presented in Figure 4.

### Selective serotonin and norepinephrine reuptake inhibitors (SSNRI)

As mentioned above, modern theories regarding depression pathology postulate that both norepinephrine and serotonin are involved in the onset of antidepressant mechanisms. Compounds with dual actions at serotonergic and noradrenergic systems have been developed, hoping that they could be effective in circumstances in which SSRI are ineffective.^10^

### Venlafaxine

Venlafaxine (*R,S*-1-[2-(dimethylamino)-1-(4-methoxyphenyl)ethyl]cyclohexanol) (VEN) is an antidepressant of the SSNRI class, used in the treatment of major depressive disorder, panic disorder, general anxiety disorder and social phobia.^32^

**Figure 5:**
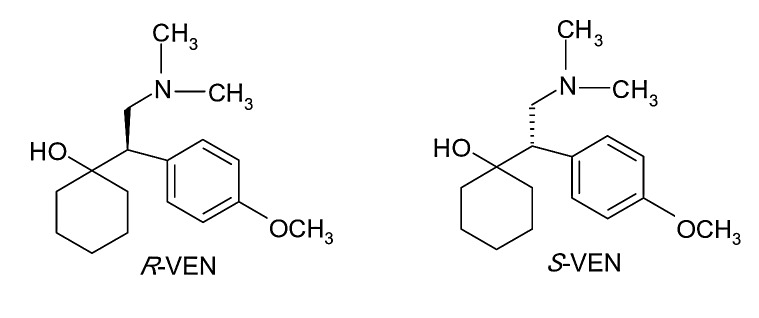
Structure of SER diastereomers

It is used in therapy as a racemic mixture, both enantiomers exhibiting useful pharmacological activity in the treatment of depression, but interacting differently with the neurotransmitters.^33^ The chemical structure of VEN enantiomers is presented in Figure 5.

*R*-VEN is a potent inhibitor of both serotonin and norepinephrine reuptake, while the *S*-VEN is more selective in inhibiting serotonin reuptake.^33^

*S*-VEN is a more potent *in vitro* inhibitor of CYP2D6 than *R*-VEN and *S*-VEN is preferentially metabolized by human CYP2D6 at therapeutic concentrations, with the opposite situation occurring at high concentrations.^34^

VEN is metabolized mainly by the CYP-450 system in the liver to O-desmethylvenlafaxine (ODVEN), N-desmethylvenlafaxine (NDVEN) and N,O-didesmethylvenlafaxine (DDVEN), active metabolites which are also chiral substances. O-Demethylation of racemic VEN to ODVEN is the predominant route of metabolism in humans, while N-demethylation of the alkylamino side chain to NDVEN and the loss of both the O-methyl and of the N-methyl groups to form DDVEN are considered minor routes. Among these, the O-demethyl derivative retains pharmacological activity comparable with the parent drug, while the N-desmethyl-derivative is also active but is a weaker inhibitor of serotonin and norepinephrine uptake than venlafaxine.^35^ The *R*- and *S*-enantiomers of the metabolites retain the properties of the parent drug regarding the potency of the inhibition of serotonin and norepinephrine respectively.^34^

### Duloxetine

Duloxetine (*(+)-S*-N-methyl-3-naphthalene-1-yloxy-3-thiophen-2-ylpropan-1-amine) (DLX) is an SSNRI used used for major depressive disorder, generalized anxiety disorder, fibromyalgia and neuropathic pain.^36^ In addition to the treatment of psychotic disorders it can also be used for treating other symptoms such as urinary incontinence.^37^

**Figure 6:**
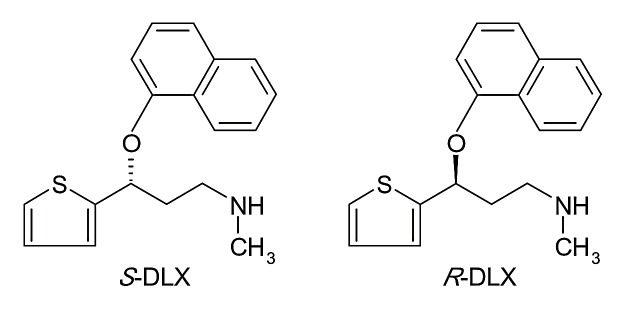
Structure of PAR diastereomers

It possesses an asymmetric carbon atom, and is used in therapy as a pure enantiomer, namely *S*-DLX. The chemical structure of DLX enantiomers is presented in Figure 6.

Both enantiomers of DLX are norepinephrine and serotonin reuptake inhibitors, although the *S*-enantiomer was found to be twice as active as the *R*-enantiomer, and subsequently introduced in therapy as a single-form enantiomer.^38^

## Conclusion

Modern antidepressant drugs (SSRIs and SSNRIs) have one or more chiral centers in their structures; their enantiomers differing from one another markedly with regard to pharmacodynamic and pharmacokinetic properties.

When using a racemic mixture differences between enantiomers with regard to absorption, protein binding, clearence, stereoselective metabolism and association of a particular adverse effect with one of the enantiomers can be observed. The use of pure enantiomers proposes potential advantages: reduction of the total amount of the administered drug; an improved therapeutic index through increased receptor selectivity and potency; reduced adverse effects; a decrease inter-individual variability in response and decreased potential for drug interactions.

The experience with *S*-CIT and *R*-FLX highlights the potential differences between enantiomers of a particular chiral drug and the need to consider pure enantiomer formulations of a previously racemic drug from case to case. Each enantiomer of a chiral drug have its own particular pharmacologic profile, and pure enantiomer formulations of a drug may possess different properties than the racemic formulation of the same drug in a chiral environment.

## Ethical Issues

Not applicable

## Conflict of Interest

The authors declare no conflict of interests.
