# Erratum to: Chirality of Modern Antidepressants: An Overview

**DOI:** 10.15171/apb.2018.041

**Published:** 2018-06-19

**Authors:** Monica Budău, Gabriel Hancu, Aura Rusu, Melania Cârcu-Dobrin, Daniela Lucia Muntean

**Affiliations:** ^1^Department of Pharmaceutical Chemistry, Faculty of Pharmacy, University of Medicine and Pharmacy from Tîrgu Mureș, Tîrgu Mureș, Romania; ^2^Department of Analytical Chemistry and Drug Analysis, Faculty of Pharmacy, University of Medicine and Pharmacy from Tîrgu Mureș, Tîrgu Mureș, Romania

In the article entitled “Chirality of modern antidepressants: an overview” which appeared in Adv Pharm Bull. 2017;7(4):495-50, the figure captions were offset in the published version of the article. The correct titles for Figure 2-6 are the following:

Figure 2. Structure of CIT enantiomers (not Stereoselective FLX metabolism)

Figure 3. Structure of SER diasteromers (not Structure of CIT enantiomers)

Figure 4. Structure of PAR diastereomers (not S-CIT metabolism)

Figure 5. Structure of VEN enantiomers (not Structure of SER diasteromers)

Figure 6. Structure of DLX enantiomers (Structure of PAR diastereomers)

The authors would like to apologize for any inconvenience this may have caused.

